# Maternal experience of intimate partner violence, maternal depression, and parental stress are not associated with child telomere length in Bangladesh

**DOI:** 10.1038/s41598-025-90505-2

**Published:** 2025-03-12

**Authors:** Diego Figueroa, Md. Mahfuz Al Mamun, Da Kyung Jung, Gaoge Li, Sophia T. Tan, Farheen Jamshed, Zachary Butzin-Dozier, Andrew N. Mertens, Jue Lin, Helen O. Pitchik, Kausar Parvin, Alexis Silvera, Lia C. H. Fernald, Benjamin F. Arnold, Shahjahan Ali, Abul K. Shoab, Syeda Luthfa Famida, Salma Akther, Md. Ziaur Rahman, Md. Saheen Hossen, Palash Mutsuddi, Mahbubur Rahman, Leanne Unicomb, Patricia Kariger, Christine P. Stewart, Alan E. Hubbard, Jade Benjamin-Chung, Firdaus S. Dhabhar, Stephen P. Luby, John M. Colford, Ruchira Tabassum Naved, Audrie Lin

**Affiliations:** 1 Division of Epidemiology and Biostatistics, School of Public Health, University of California, Berkeley, Berkeley Way West, 2121 Berkeley Way, #5302, Berkeley, CA 94704 USA; 2 Maternal and Child Health Division, International Centre for Diarrhoeal Disease Research, Bangladesh (icddr,b), Dhaka, 1212 Bangladesh; 3 Division of Infectious Diseases and Geographic Medicine, Stanford University, Y2E2, MC #4205, 473 Via Ortega, Stanford, CA 94305 USA; 4 Department of Biochemistry and Biophysics, University of California, San Francisco, 600 16th St, San Francisco, CA 94158 USA; 5 Division of Community Health Sciences, School of Public Health, University of California, Berkeley, Berkeley Way West, 2121 Berkeley Way, #5302, Berkeley, CA 94704 USA; 6 Francis I. Proctor Foundation, University of California, San Francisco, California, 95 Kirkham Street, San Francisco, CA 94143 USA; 7 Environmental Health and WASH, Health System and Population Studies Division, International Centre for Diarrhoeal Disease Research, Bangladesh (icddr,b), Dhaka, 1212 Bangladesh; 8 Department of Microbiology and Environmental Toxicology, University of California, Santa Cruz, Physical Sciences Building 446, 590 Steinhart Way, Santa Cruz, CA 95064 USA; 9 Institute for Global Nutrition, University of California Davis, 3135 Meyer Hall, One Shields Avenue, Davis, CA 95616 USA; 10 Department of Epidemiology and Population Health, Stanford University, Alway Building, 300 Pasteur Drive, Stanford, CA 94305 USA; 11 Department of Psychiatry and Behavioral Sciences, Department of Microbiology & Immunology, Sylvester Comprehensive Cancer Center, Miller School of Medicine, University of Miami, 1120 NW 14th Street, Miami, FL 33136 USA; 12 Johns Hopkins University School of Medicine, 733 N Broadway, Baltimore, MD 21205 USA; 13 School of Medicine, Oregon Health & Science University, 3181 SW Sam Jackson Park Road, Portland, OR 97239 USA; 14 School of Medicine, New York Medical College, 40 Sunshine Cottage Road, Valhalla, NY 10595 USA

**Keywords:** Intimate partner violence, Depression, Perceived stress, Telomere length, Maternal child health, Rural Bangladesh, PCR-based techniques, Epidemiology, Paediatric research, Socioeconomic scenarios, Depression

## Abstract

Shorter telomere length (TL) is associated with an increased risk for developing chronic or age-related diseases in adults. The process of telomere shortening is accelerated in response to stress and is well characterized in adult populations from high-income countries. Prior studies suggest the relationship between stress, shorter TL, and disease risk initiates in early life. Nested within the WASH Benefits Bangladesh trial, we examined associations between parental stressors, including maternal exposure to intimate partner violence (IPV), maternal depressive symptoms, and parental perceived stress, and child TL in rural Bangladesh. We measured whole blood relative TL in 660 children at median age 14 months and 702 children at median age 28 months. We estimated mean differences between the 25th and 75th percentile or absence and presence of each exposure using generalized additive models. IPV during pregnancy was associated with more TL attrition between 14 and 28 months (− 0.32 (95% CI − 0.64, − 0.01), p-value 0.05). This association was not significant after correction for multiple comparisons. Other parental psychosocial stressors were not associated with child TL outcomes at 14 or 28 months of age in rural Bangladesh. Telomere biology during early-life development may vary across settings.

**Supplementary Information:**

The online version contains supplementary material available at 10.1038/s41598-025-90505-2.

## Introduction

Intimate partner violence (IPV) against women is an urgent public health issue that has both short- and long-term health implications, particularly for maternal and child health. Globally, an estimated 27% of reproductive aged women have experienced physical and/or sexual IPV during their lifetime and 13% in the past 12 months^1^. This violence starts early, with 24% of the adolescent girls aged 15–19 years and 26% of the young women aged 20–24 years having already experienced IPV at least once since age 15 globally^1–3^. A recent World Health Organization report revealed a higher lifetime prevalence of physical and/or sexual intimate partner violence (IPV) in low-income countries (37%), including Bangladesh (50%), compared to high-income settings (e.g., 16–23% in Europe)^4^.

Consequences of IPV include higher rates of chronic illnesses, depression, anxiety, phobias, and suicidal thoughts and attempts^5^. One study in rural Bangladesh found a positive dose-response relationship between the severity of psychological, physical, and sexual IPV and an elevated risk for major depressive episodes in women^6^. Those who experience IPV during pregnancy are at a higher risk for adverse birth outcomes such as preterm birth^7^. IPV during the postpartum period has also been associated with increased maternal perceived stress and depression^8^.

The health effects of psychosocial stressors like IPV, perceived stress, and maternal depression during pregnancy may transcend generations through hormonal, immunological, and metabolic interactions with the developing fetus. *In utero* and/or postpartum exposure to such stressors increase the risk for impaired emotional, neurological, and behavioral development^9–12^ as well as stunting during childhood^13,14^. Further evidence suggests that intrauterine and postpartum exposure to psychological stressors have molecular and genetic interactions which predispose infants to increased disease risk and susceptibility later in life; a process known as “fetal programming”^15,16^. Studies have observed a strong correlation between prenatal stress and the development of obesity and other metabolic disorders later in life^17–19^. Given the complex and generational implications, the prevalence of IPV poses significant risks to the health of women and children with the potential to incur multi-generational health consequences.

In recent decades, telomeres have emerged as a viable metric for measuring the relationship between stress and health outcomes. Telomeres are double-stranded, non-coding, repeated DNA sequences (TTAGGG in humans) that serve as the protective ends of chromosomes. Telomeres shorten with each cellular division and prevent loss of coding DNA during replication^20^. Telomere length (TL) naturally shortens with age^21^, decreasing the fastest in the first years of life, followed by a constant steady state between 4 years of age and young adulthood until finally reaching a gradual rate of attrition in later adulthood^22^. Longer TL is associated with better growth during early childhood^23^. TL shortening beyond a critical threshold initiates the cascade of cellular apoptosis, tissue damage, and a myriad of physiological responses resulting in a diseased state^24^. Shorter TL in adults has been implicated in numerous non-communicable diseases such as Type 2 diabetes mellitus^25^, Alzheimer’s disease^20^, certain types of cancer^26^, myocardial infarction^27,28^, and stroke^28^. In addition to genetics, lifestyle, and natural aging, environmental and psychological stress may impact the rate of telomere shortening throughout one’s lifespan, beginning as early as *in utero*^29,30^. Thus, telomere biology during development may be a potential mechanistic pathway through which early-life psychosocial stress influences subsequent health outcomes.

Research suggests that TL at birth is a strong predictor of later TL, suggesting the importance of prenatal factors that shape child TL during gestation^31^. Psychosocial and environmental perturbations within this developmental window, such as exposure to childhood trauma and institutionalization, have been associated with shorter child telomere length^29,32,33^. Additionally, young children affected by family violence, suicide, or incarceration, especially those who witnessed family violence, were observed to have significantly shorter telomeres^34^. These findings illustrate how adverse events in early life are related to TL in infancy and early childhood, when the rate of telomere attrition is most dynamic^35^.

The extent that early life stressors, such as *in utero* exposure to maternal stress, IPV, and depression, affect TL in young children and later in life are not well understood. Research examining the relationship between intrauterine exposure to maternal stress, depression, and IPV and initial TL currently yields mixed results and lacks coverage in low-income settings. Some studies observed that maternal exposure to psychosocial stressors was correlated with decreased child TL in newborns^36–41^, at age 4 years^42^, at ages 6 to 16 years^43^, and in young adults^44^, while others found no significant association between maternal stressors during pregnancy and newborn TL^45,46^ or at age 2 years^45^. Discrepancies between these findings are likely attributable to inconsistencies across TL pre-analysis and analysis methods, selection and adjustment for confounders, and challenges establishing temporality as most TL studies have been conducted in adult populations and analyzed retrospectively. Additionally, research regarding paternal psychological stress is lacking in the literature, except one study that found paternal post-traumatic stress disorder (PTSD) prior to conception was not associated with child TL at age 4 years^42^. The majority of telomere research has been conducted in middle- or high-income countries, highlighting the research gap in low-income regions where more abundant stressors may impact TL. Because of the mixed and limited evidence, the aim of this study was to elucidate the relationship between maternal exposure to IPV, maternal depression, paternal perceived stress, and child TL during the first two years of life in a population living in rural Bangladesh.

## Results

### Methods summary

**Fig. 1:**
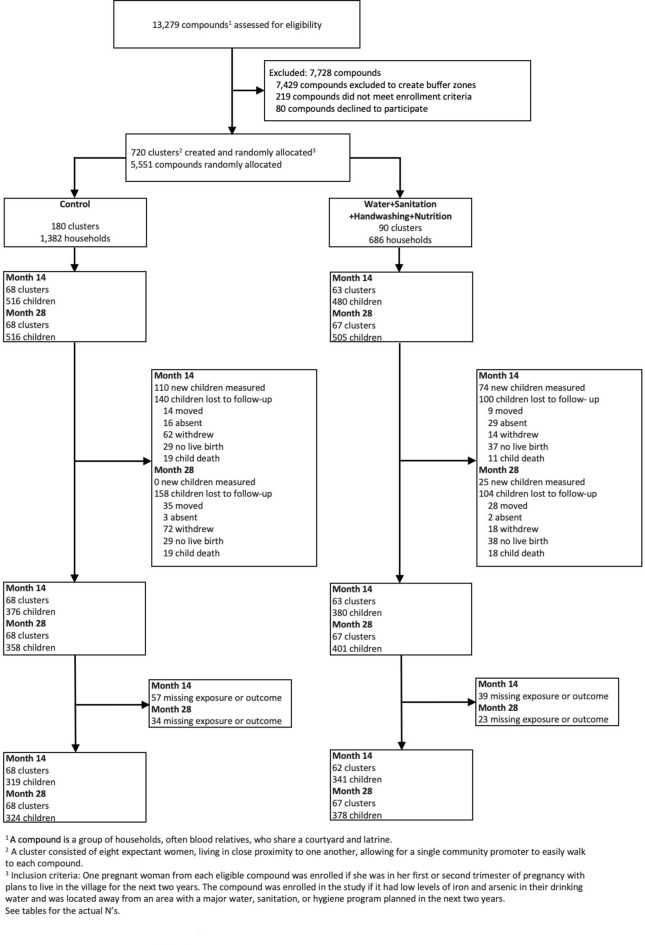
Participant enrollment figure. Diagram of participants at each phase of the maternal intimate partner violence, stress, maternal depression, and parental perceived stress and child telomere length substudy within the WASH Benefits Bangladesh trial. Children were included if exposure and outcome measurements were available. Reasons for loss to follow up are listed.

We assessed associations of maternal exposure to IPV, maternal depressive symptoms, and maternal and paternal perceived stress with child TL at median age 14 months (Year 1) and 28 months (Year 2) in rural Bangladesh. In the WASH Benefits parent trial, 5551 pregnant women in 720 clusters were enrolled between May 2012 and July 2013. The trial included 6 intervention arms and a double-sized control arm, and this substudy only included children from the control and combined nutrition, water, sanitation, and handwashing arms. Children from the birth cohort of the two arms were included in the current study’s analyses if maternal IPV (physical, emotional, or sexual) measured by a modified version of the Conflict Tactics Scale 2; maternal depression measured by the Center for Epidemiologic Studies Depression Scale Revised; parental perceived stress measured by the Perceived Stress Scale; and child whole blood relative TL were available. Either the exposure or outcome was missing from 96 (12.7%) children at Year 1 and 57 (7.5%) children at Year 2. Therefore, 660 children at Year 1 (median age 14 months, Quartile 1, Quartile 3 (Q1, Q3): 13, 16) and 702 children at Year 2 (median age 28 months, Q1, Q3: 27, 30) were included (Fig. 1). For the statistical analyses, mean differences were estimated between the 25th and 75th percentile or between the absence and presence of each exposure using generalized additive models. Detailed protocols are included in Methods below.

### Child and maternal characteristics

Of the enrolled children, 51% were female. The median length-for-age Z-score was − 1.28 (Q1, Q3: − 1.98, − 0.53) at age 3 months, − 1.41 (Q1, Q3: − 2.09, − 0.78) at age 14 months, and − 1.54 (Q1, Q3: − 2.25, − 0.94) at age 28 months (Table 1). Past 7-day prevalence of diarrhea at Year 2 (8%) was lower than at Year 1 (14%). We observed a coefficient of variation in child TL of 0.168 at Year 1 and 0.159 at Year 2. The correlation coefficient between the two TL z-scores was 0.625 (95% CI 0.572, 0.674, p-value < 2.2e-16). The median age of the women at enrollment was 23 (Q1, Q3: 20, 27) years, with a median height of 150.5 cm (Q1, Q3: 147.1, 153.9). The median years of education for the women was 7 years (Q1, Q3: 4, 9).

**Table 1:** Characteristics of participants.

			*n* (%) or median (Q1, Q3^1^)
Child		Female	416 (51%)
	Telomere length at Year 1	T/S ratio^2^	1.42 (1.28, 1.56)
	Telomere length at Year 2	T/S ratio^2^	1.43 (1.29, 1.58)
	Change in telomere length between Year 1 and Year 2	T/S ratio^2^	0.04 (− 0.22, 0.25)
	Anthropometry (3 months, Year 1)	Length-for-age Z score	− 1.28 (− 1.98, − 0.53)
		Weight-for-age Z score	− 1.18 (− 1.83, − 0.5)
		Weight-for-length Z score	− 0.25 (− 1.1, 0.44)
		Head circumference-for-age Z score	− 1.72 (− 2.38, − 1.01)
	Anthropometry (14 months, Year 1)	Length-for-age Z score	− 1.41 (− 2.09, − 0.78)
		Weight-for-age Z score	− 1.3 (− 1.98, − 0.68)
		Weight-for-length Z score	− 0.89 (− 1.61, − 0.25)
		Head circumference-for-age Z score	− 1.79 (− 2.39, − 1.19)
	Anthropometry (28 months, Year 2)	Length-for-age Z score	− 1.54 (− 2.25, − 0.94)
		Weight-for-age Z score	− 1.55 (− 2.09, − 0.9)
		Weight-for-length Z score	− 1 (− 1.59, − 0.37)
		Head circumference-for-age Z score	− 1.78 (− 2.37, − 1.22)
	Diarrhea (14 months, Year 1)	Caregiver-reported 7-day recall	104 (14%)
	Diarrhea (28 months, Year 2)	Caregiver-reported 7-day recall	56 (8%)
Mother		Age (years)	23 (20, 27)
	Anthropometry at enrollment	Height (cm)	150.52 (147.1, 153.91)
	Education	Schooling completed (years)	7 (4, 9)
	Depressive symptoms at Year 1	CESD-20^3^ score	10 (6, 16)
	Depressive symptoms at Year 2	CESD-20^3^ score	10 (5, 17)
	Perceived stress at Year 2	Perceived stress scale score	14 (10, 18)
	Any type of intimate partner violence	Any lifetime exposure	398 (56%)
	Physical violence	Any lifetime exposure	324 (46%)
	Emotional violence	Any lifetime exposure	265 (37%)
	Sexual violence	Any lifetime exposure	159 (22%)

^1^Q1, Q3 = Quartile 1, Quartile 3 (25th percentile, 75th percentile).

^2^The unit for relative telomere length is the T/S ratio. Telomere length was measured by quantitative PCR (qPCR), a method that determines relative telomere length by measuring the factor by which each DNA sample differs from a reference DNA sample in its ratio of telomere repeat copy number (T) to single-copy gene copy number (S).

^3^CESD-20 = Center for Epidemiologic Studies Depression Scale Revised.

The enrollment characteristics included in this table were selected to provide context for our study population and to help frame our results. The table is not intended to summarize all covariates, but rather to highlight factors we consider most relevant to TL. Specifically, we focused on variables that are known to be associated with child telomere length, such as growth^23^.

### IPV and TL

**Fig. 2:**
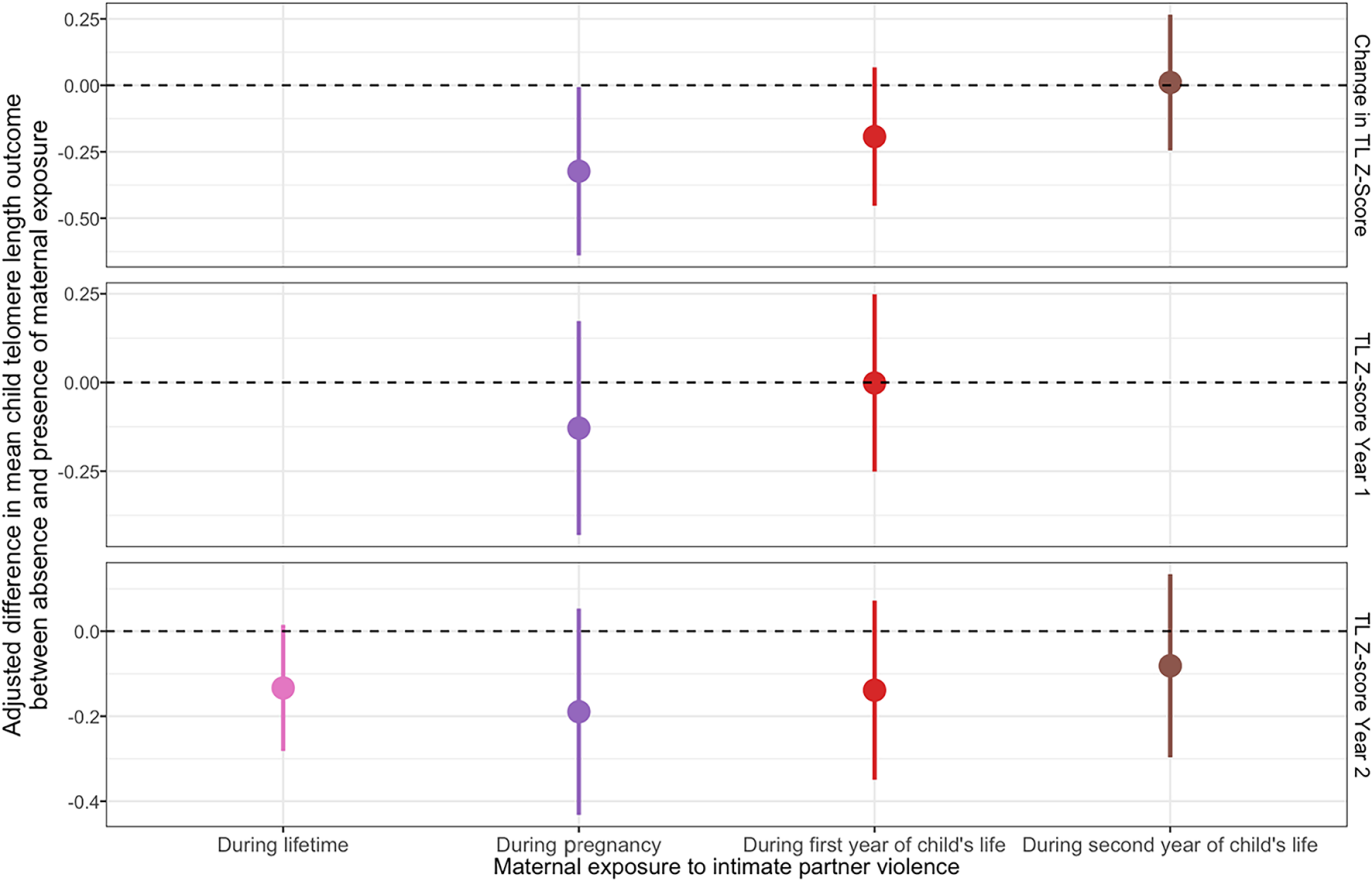
A visual summary of the associations between maternal exposure to intimate partner violence and child telomere length. Each dot represents the mean difference in predicted telomere length outcomes in children whose mothers reported exposure to intimate partner violence (IPV) and children whose mothers reported no exposure to IPV. Vertical lines represent the 95% confidence intervals. All results were adjusted for pre-specified and pre-screened covariates: child sex, child birth order, mother’s age, mother’s height, mother’s education, household food security, number of children < 18 years old in the household, number of people living in the compound, month of exposure and outcome measurement, treatment arm, distance (in minutes) to the primary water source, household materials (wall, floor, roof), asset-based household wealth (electricity, wardrobe, table, chair or bench, khat, chouki, working radio, working black/white or color television, refrigerator, bicycle, motorcycle, sewing machine, mobile phone, land phone, number of cows, number of goats, number of chickens).

Of the women surveyed in the study, over half (*n* = 398, 56%) reported having had exposure to any type of IPV during their lifetime (Table 1). We did not detect a statistically significant association (defined as *p* < 0.05) between any lifetime exposure to IPV and any child TL outcome at Year 1 or Year 2 (Table 2). Similarly, IPV exposure during pregnancy was not significantly associated with child TL at Year 1 or TL at Year 2 (Table 2). IPV during pregnancy was negatively associated with the change in TL Z-score between Year 1 and Year 2 (− 0.32, 95% CI − 0.64, − 0.01, p-value 0.05; Table 2). However, the association was not significant (defined as *p* < 0.2) after False Discovery Rate (FDR) correction using the Benjamini-Hochberg procedure (Supplementary Table 1). There was no association between maternal exposure to IPV during the first year of a child’s life and child TL at Year 1 nor between maternal exposure to IPV during the second year of a child’s life (measured at Year 2) and child TL at Year 2 (Table 2). Overall, our point estimates for associations between each IPV-TL exposure-outcome pair assessed, including the one significant association, consistently reflected a negative direction in point estimates between maternal IPV and child TL, except two contrasts that yielded zero to near-zero point estimates (maternal exposure to IPV between birth and Year 1 and child TL at Year 1, maternal exposure to IPV between Years 1 and 2 and child TL at Year 2) (Table 2; Fig. 2).

**Table 2:** Association between maternal exposure to intimate partner violence and child telomere length.

Intimate partner violence (IPV)	Outcome	*N*	Outcome, with exposure v. without exposure			
			Adjusted			
			Predicted outcome if not exposed	Predicted outcome if exposed	Predicted outcome difference (95% CI)	*P*-value
Lifetime exposure to IPV Year 2	Telomere length Z-score Year 2	678	0.3	0.17	− 0.13 (− 0.28, 0.02)	0.08
Exposure to IPV during pregnancy	Telomere length Z-score Year 1	291	0	− 0.13	− 0.13 (− 0.43, 0.17)	0.41
	Telomere length Z-score Year 2	366	0.03	− 0.16	− 0.19 (− 0.43, 0.05)	0.13
	Change in telomere length Z-Score	279	0.26	− 0.06	− 0.32 (− 0.64, − 0.01)	0.05
Exposure to IPV between birth and Year 1	Telomere length Z-score Year 1	302	− 0.07	− 0.08	0 (− 0.25, 0.25)	0.99
	Telomere length Z-score Year 2	381	− 0.11	− 0.25	− 0.14 (− 0.35, 0.07)	0.2
	Change in Telomere length Z-Score	287	0.26	0.06	− 0.19 (− 0.45, 0.07)	0.15
Exposure to IPV between Year 1 and Year 2	Telomere length Z-score Year 2	381	− 0.06	− 0.14	− 0.08 (− 0.3, 0.13)	0.47
	Change in telomere length Z-Score	287	0.17	0.18	0.01 (− 0.24, 0.27)	0.94

N, Predicted Outcome if not exposed, and Predicted Outcome if exposed are from the adjusted generalized additive model analyses.

Adjusted for pre-specified and pre-screened covariates: child sex, child birth order, mother’s age, mother’s height, mother’s education, household food security, number of children < 18 years old in the household, number of people living in the compound, month of exposure and outcome measurement, treatment arm, distance (in minutes) to the primary water source, household materials (wall, floor, roof), asset-based household wealth (electricity, wardrobe, table, chair or bench, khat, chouki, working radio, working black/white or color television, refrigerator, bicycle, motorcycle, sewing machine, mobile phone, land phone, number of cows, number of goats, number of chickens).

### Depressive symptoms and TL

The median Center for Epidemiologic Studies Depression Scale Revised (CESD-20) score was 10 at Year 1 (Q1, Q3: 6, 16) and Year 2 (Q1, Q3: 5,17) (Table 1). No association was detected between the maternal CESD-20 score measured at Year 1 and concurrent child TL or between CESD-20 at Year 1 and child TL at Year 2 (Table 3). Similarly, there was no significant association between CESD-20 at Year 1 and change in TL (Table 3). Finally, no association was observed between CESD-20 at Year 2 and concurrent TL (Table 3). Though the point estimates for the association between most maternal depression-TL exposure-outcome pairs were positive, there was no maternal depression-TL association that was statistically significant.

**Table 3:** Association between maternal depression and child telomere length.

Maternal depressive symptoms	Outcome	*N*	25th percentile	75th percentile	Outcome, 75th percentile v. 25th percentile or with exposure v. without exposure			
					Adjusted			
					Predicted Outcome at 25th Percentile or if Not Exposed	Predicted Outcome at 75th Percentile or if Exposed	Predicted Outcome Difference (95% CI)	*P*-value
Continuous maternal depressive symptoms Year 1	Telomere length Z-score Year 1	637	6	16	0.09	0.15	0.07 (− 0.03, 0.16)	0.18
	Telomere length Z-score Year 2	692	6	16	0.2	0.25	0.05 (− 0.04, 0.13)	0.29
	Change in Telomere length Z-Score	543	6	16	0.26	0.25	− 0.01 (− 0.11, 0.1)	0.87
Binary maternal depressive symptoms year 1	Telomere length Z-score Year 1	637	--	--	0.1	0.21	0.11 (− 0.06, 0.28)	0.22
	Telomere length Z-score Year 2	692	--	--	0.21	0.31	0.1 (− 0.06, 0.27)	0.22
	Change in Telomere length Z-Score	543	--	--	0.26	0.24	− 0.02 (− 0.21, 0.17)	0.82
Continuous maternal depressive symptoms year 2	Telomere length Z-score Year 2	679	5	17	0.19	0.22	0.03 (− 0.06, 0.11)	0.57
Binary maternal depressive symptoms year 2	Telomere length Z-score Year 2	679	--	--	0.19	0.27	0.08 (− 0.09, 0.24)	0.36

N, Predicted Outcome at 25th percentile of exposure distribution or if not exposed, and Predicted Outcome at 75th percentile of exposure distribution or if exposed are from the adjusted generalized additive model analyses.

Adjusted for pre-specified and pre-screened covariates: child sex, child birth order, mother’s age, mother’s height, mother’s education, household food security, number of children < 18 years old in the household, number of people living in the compound, month of exposure and outcome measurement, treatment arm, distance (in minutes) to the primary water source, household materials (wall, floor, roof), asset-based household wealth (electricity, wardrobe, table, chair or bench, khat, chouki, working radio, working black/white or color television, refrigerator, bicycle, motorcycle, sewing machine, mobile phone, land phone, number of cows, number of goats, number of chickens).

### Perceived stress and TL

The median maternal Perceived Stress Score (PSS) score at Year 2 was 14 (Q1, Q3: 10, 18) (Table 1), similar to the reference norm among women in the United States^44^. No associations were observed between maternal perceived stress and child TL at Year 2 (Table 4). Paternal perceived stress at Year 2 also had no significant association with concurrent TL (Table 4).

**Table 4:** Association between parental perceived stress and child telomere length.

Parental perceived stress	Outcome	*N*	25th percentile	75th percentile	Outcome, 75th percentile v. 25th percentile			
					Adjusted			
					Predicted outcome at 25th percentile	Predicted outcome at 75th percentile	Predicted outcome difference (95% CI)	*P*-value
Maternal perceived stress	Telomere length Z-score Year 2	680	10	18	0.21	0.18	− 0.04 (− 0.14, 0.07)	0.51
Paternal perceived stress	Telomere length Z-score Year 2	498	14	21	0.2	0.33	0.13 (− 0.05, 0.32)	0.16

N, Predicted Outcome at 25th percentile of exposure distribution, and Predicted Outcome at 75th percentile of exposure distribution are from the adjusted generalized additive model analyses.

Adjusted for pre-specified and pre-screened covariates: child sex, child birth order, mother’s age, mother’s height, mother’s education, household food security, number of children < 18 years old in the household, number of people living in the compound, month of exposure and outcome measurement, treatment arm, distance (in minutes) to the primary water source, household materials (wall, floor, roof), asset-based household wealth (electricity, wardrobe, table, chair or bench, khat, chouki, working radio, working black/white or color television, refrigerator, bicycle, motorcycle, sewing machine, mobile phone, land phone, number of cows, number of goats, number of chickens).

### Post-hoc analyses

The Pearson correlations between maternal IPV, maternal depressive symptoms and parental perceived stress were all weakly to moderately positive, while the correlations with TL were negative for the TL change, and inconsistent with TL at Year 1 and Year 2, with only lifetime maternal IPV being negatively correlated with TL at both timepoints, as would be expected with a negative marker of maternal wellbeing (Supplementary Table 5). The correlations were consistent with the effect direction with TL, with all but one matching (Maternal CESD and TL at Year 2), though the correlation was weak and not significant when tested.

We conducted a post-hoc analysis by IPV type and frequency of exposure. Lifetime prevalence of exposure to physical, emotional, and sexual violence was 46%, 37%, and 22% respectively (Table 1). Maternal exposure to physical, emotional, and sexual violence were not significantly associated with any child TL outcome (Supplementary Table 4). There were no significant associations between the frequency of each type of IPV and child TL outcomes (Supplementary Fig. 1).

## Discussion

Here, we report on the relationship between parental psychosocial challenges and child TL during the first two years of life in rural Bangladesh. We did not detect consistent, statistically significant associations between maternal exposure to IPV, maternal depressive symptoms, or parental perceived stress and child TL or TL attrition at 1 or 2 years of age. Maternal exposure to IPV during pregnancy was associated with greater negative change in child TL between Year 1 and Year 2, but this was not significant after FDR correction. This result is in coherence with a previous study in Hong Kong that observed that maternal IPV exposure any time before birth was associated with shorter newborn TL^37^. Given the consistent, negative direction of point estimates across our exposure-outcome pairs of maternal IPV-child TL but lack of statistical significance, further study will be needed to assess this potential inverse relationship.

The vast majority of our results indicated no statistically significant association and are not consistent with existing studies that link maternal psychosocial stress with shorter child TL in high-income countries^36,39–41,43,44^. Maternal perceived stress in Germany^36^ and the United States^39^ were associated with shorter TL in newborns. Also in the U.S., maternal PTSD but not paternal PTSD was associated with shorter child TL at age 4 years^42^ and maternal perceived stress and depressive symptoms were associated with shorter TL in child ages 6–16 years^43^. Maternal depression was associated with shorter TL in newborns in Singapore^40^, newborns and at age 1 year in South Korea^41^, and in girls aged 10–14 years in the U.S^44^. Instead, our results align with a large Finnish study that found no correlation between prenatal stress and newborn TL^46^, as well as a South African study that reported no association between maternal exposure to IPV, depression, PTSD, or childhood trauma and child TL as newborns or at age 2 years^45^. The present study is the first to examine these links in a rural Bangladesh study population aged 1 and 2 years. Telomeric processes are highly dynamic during this time with rapid lengthening and shortening^35^, which may explain why our results differ from studies where newborns’ or older children’s TL were examined. Overall, our findings indicate that telomere biology and attrition in early-life development may vary across settings.

Several elements may account for the discrepancies between our findings and existing research that demonstrates an inverse association between maternal psychosocial stress and child TL. First, most similar studies have been conducted in high-income countries exposed to considerably less complex environmental stress profiles compared to low-income settings within rural Bangladesh. This context increases the risk for exogenous stressors that may act as unmeasured confounders. The sample population live in a low-resource setting, where they experience continuous exposure to a diverse array of stress-inducing factors that affect maternal health, infant growth, and immune system development that remain unmeasured in this study—for example, maternal infection during pregnancy or other factors associated with low socioeconomic status. Chronic exposure to many forms of adversity early in life can undermine biological resiliency against stress^47,48^. These unmeasured adverse events may be more pervasive and have stronger effects on early-life TL than the measured parental psychosocial stressors, resulting in no observation of the hypothesized associations. Furthermore, TL shortening processes may serve a different functional role in high adversity, low-resource contexts compared to high-resources contexts—where exposure to stress is generally associated with TL shortening^36,37,39–41,43,44^. For example, our previous WASH Benefits trial in Bangladesh found that the combined effects of safe water, sanitation, and handwashing stations in addition to nutritional supplementation for children led to improved growth, but also shorter TL compared to the control group^49^. This finding demonstrates a potentially adaptive role of telomere attrition in early life, where the energy associated with maintaining long telomeres is “sacrificed” to promote rapid growth and immune system development, rather the attrition being a consequence of stress^23,50^. In a low-resource setting, there may be interactions between the adaptive role of telomere shortening^35^ and telomere shortening from stressors, meaning associations observed in high-resource settings may not hold true.

Social dynamics that differ between high- and low-resource regions may also mask the prevalence and effects of psychosocial stressors^6^. Cross-cultural differences in perceptions of IPV and depression have been well documented and offer another plausible explanation for why our results diverge from findings in high-income settings based on social and cultural characteristics^51^. For example, considerable research conducted in Bangladesh suggests a significant degree of social acceptability in “justified” or “appropriate” levels of IPV^6,52,53^. A normative perception of IPV in particular communities may affect one’s internalization of such events^54^, influencing mental health, and thus minimizing negative secondary health outcomes^6^. Another study in Bangladesh revealed that women who experienced more severe acts of IPV were more likely to report these instances compared to women who experienced less severe forms^55^. If this is true in our dataset, it would lead to underreporting of IPV. If there is an underlying relationship between both severe and less severe IPV and TL, this underreporting would lead to an underestimate of the associations found in the present analysis. This brings forth the limitation of using dichotomous measures of IPV exposure instead of classification based on severity, which may be more accurate^6^. Hence, we conducted post-hoc analyses with categorical variables to represent exposure to varying frequencies of each type of IPV. Though no results were significant, we highlight the importance of parsing out the nuances of IPV by frequency and type of violence. Finally, there are known mediators of the relationship between maternal stress and newborn TL, such as maternal psychological resiliency, which play a protective role against these stressors^56^. The effect of social perception, variation in survey and analytical methods, and stress mediation through psychosocial resilience may mask or attenuate the true prevalence and effect, if any, of maternal IPV, stress, and depression on child TL in our study. Future analysis should account for these social factors, measure perceived severity of IPV, and include markers of systemic inflammation. Accounting for these social and protective aspects will aid in better understanding their role, if any, in the pathway between psychological stress and the downstream physiological response.

Another difference between the present work and previous studies relates to the timing and method of sample collection for TL measurement. The majority of studies measured TL from umbilical cord blood^36,37,39,41,45,46^. Whether they utilized leukocyte DNA^39,41,46^ or all genomic DNA^36,37,40,45^, overall, umbilical cord derived TL may reflect a different TL profile than peripheral whole blood derived TL because over 80% of cord blood cells are leukocytes and most cells are hematopoietic or progenitor cells^57^. We measured TL in whole blood, which includes a heterogeneous sample of cell types, resulting in a composite measurement of TL. Evidence indicates whole blood TL can serve as a proxy for TL in many tissues, as TLs were positively correlated within an individual^58^. In fact, whole blood may be preferred since TL in whole blood is more dynamic and likely to reflect the biochemical and molecular impact caused by prenatal stress. Of note, however, two studies observed consistent results between newborn umbilical cord TL and early childhood peripheral blood TL^41,45^. Furthermore, because we measured telomere length in whole blood samples, we were not able to account for potential variability in telomere length assessments due to heterogeneity in blood cell composition across individuals. We recommend that future studies include measurements of blood cell composition. In terms of timing, it is possible that maternal stress and IPV exposure may be associated with newborn TL but not child TL at ages 1 and 2 years. A meta-analysis showed that the magnitude of the association between early adversity and child TL was initially strong but decreased over time since initial exposure^59^, potentially due to telomerase activity^60^. However, among children in South Korea, significant associations between prenatal maternal depression and anxiety and child TL were present in newborns and remained consistent at age 1 year^41^. Two other studies reported an association between maternal prenatal stress exposure and shorter TL in late childhood and early adulthood^43,44^, suggesting that TL attrition associated with maternal prenatal stress exposure may persist until later in life. Future longitudinal studies are needed to better understand patterns over time by documenting maternal exposures and following TL at multiple time points extending into adolescence and adulthood.

Finally, differences in pre-analytic and analytic methods used may have also led to inconsistencies between study results. The lack of standardized laboratory methods for TL quantification may lead to variable precision^61^. Most studies, including this study, used qPCR^36,37,44–46^, which has been shown to generate T/S ratios that are, at times, not comparable across different studies due to differences in laboratories and DNA extraction methods^61,62^. Yet, this method is still quite reproducible, with a correlation r value of > 0.9, making it less likely to be a major contributor to discrepancies between studies^63^. Studies, however, vastly differ in the covariates included in their statistical models. Previous studies adjusted for maternal covariates only or child covariates only, with some including covariates related specifically to pregnancy^46^. This study accounts for potential confounders and biases that were not addressed in previous studies such as child birth order, household size, household food insecurity, and other child, maternal, and household characteristics that may impact familial dynamics and child TL. The full list of covariates adjusted for are found in the footnote of Table 2.

This study advances the field by addressing a critical knowledge gap in the literature pertaining to the study population, both in terms of setting and participant age. We also present a large sample from a well-characterized cohort of children. The sample size is comparable to or larger than existing studies. In addition, we tested and controlled for a more comprehensive set of pre-specified child, mother, and household covariates, while other studies focused on covariates related to only the child or mother.

There are several limitations to our study. First, maternal exposure to IPV during pregnancy, the first two years of their child’s life, and their lifetime was retrospectively assessed when their child was 2 years old, which may be subject to recall bias. However, IPV often follows a recurring pattern where women who experienced IPV in the past are more likely to experience it in the present^2^, and a pattern is more likely to be recognized or recalled than a standalone event. Second, we did not measure child TL at birth, which limits our interpretation of our results. We cannot conclude whether there is no relationship between parental psychosocial stressors and child TL at all, or if there is a relationship at birth that declines by the age of one. Third, we did not measure parent TL, so we were not able to assess any relationships between parent and child TL on a potential causal pathway. Additionally, we measured child TL using qPCR, which has a larger measurement error than Southern blots, which is the gold standard^64^. However, both qPCR and Southern blot techniques for TL measurement have correlation r values of > 0.9, indicating they are both reproducible techniques^63^. Also, our large sample size lowers the risk of this measurement error. Especially in a low-resource setting, qPCR is more accessible as it is faster, cheaper, and requires less DNA^61^. Finally, the FDR correction methods may have been overly conservative for the exposures included in this study, which could be associated with each other. We addressed this limitation by interpreting p-values in conjunction with the consistency of the direction of point estimates. Lastly, due to the observational study design, our study results may also be affected by residual confounding.

In summary, we observed no significant associations between maternal exposure to IPV, maternal depressive symptoms, and parental perceived stress and child TL. Several potential explanations exist for the observed discrepancies between our findings and those of previous studies regarding the relationship between maternal stress, IPV, and child TL. As the molecular basis of early childhood stress and TL attrition is still largely unknown^65^, future studies should investigate the biological mechanisms that underlie any reported associations between maternal exposure to IPV, depression, stress, and child TL. Additionally, while several studies have focused on the relationship between maternal stress and IPV exposure and child TL, there is a paucity of studies elucidating the links between maternal depression and child TL. Future investigations should adopt longitudinal designs that track maternal exposures and child TL at multiple points from birth onward to better understand telomere biology and the intergenerational impact of parental stress.

## Methods

### Study design

The WASH Benefits Bangladesh study was a cluster-randomized, geographically matched trial with six intervention arms and one double-sized control arm. The WASH Benefits trial aimed to study the effects of water, sanitation, handwashing, and nutrition interventions on child outcomes, including telomere length, in the Gazipur, Kishoreganj, Mymensingh, and Tangail districts of rural Bangladesh^49^. This observational study was nested within the main WASH Benefits study^66^.

### Participants

In this study, participants consisted of women and their children recruited from compounds within the control and combined nutrition, water, sanitation, and handwashing (N + WSH) intervention arms of the WASH Benefits study between 31 May 2012 and 7 July 2013^67^. In rural Bangladesh, a compound is a group of households, often blood relatives, who share a courtyard and latrine^66^. One pregnant woman from each eligible compound was recruited into the study. A woman was enrolled in the study if she was in her first or second trimester of pregnancy with plans to live in the village for the next two years. The compound was enrolled in the study if it had low levels of iron and arsenic in their drinking water and was located away from an area with a major water, sanitation, or hygiene program planned in the next two years^66^. Each community health promoter delivered interventions to eight pregnant women within walking distance; women living in compounds too remote for community health promoters to reach on foot were not selected. Children *in utero* at the time of their mother’s enrollment were screened for exclusion criteria previously described^67^ and eligible children provided biological specimens. A child was included in this substudy if their mother completed the IPV and depression surveys, their parents completed the perceived stress survey, and the child had provided whole blood samples for TL measurement.

### Ethics

All participants and caregivers in the study provided written informed consent prior to study enrollment. The ethical review committees at the International Centre for Diarrhoeal Disease Research, Bangladesh (icddr, b) (PR-11063 and PR-14108), the University of California, Berkeley (2011-09-3652 and 2014-07-6561), and Stanford University (25863 and 35583) approved the study protocols^67^. All methods were performed in accordance with the relevant guidelines and regulations. The parent trial was registered at ClinicalTrials.gov (NCT01590095).

### Exposures

In this substudy, the following maternal (IPV and depressive symptoms) and maternal and paternal (perceived stress) exposures were measured through surveys during the first two years of child life. Data was collected at Year 1 (median child age of 14 months) and Year 2 (median child age of 28 months).

### Maternal exposure to IPV

We measured maternal exposure to IPV at Year 2 with a modified version of the Conflict Tactics Scale 2 (CTS2)^68^ used in the WHO Women’s Health and Life Experiences Survey^69^. The scale is the most widely used measure of IPV containing direct and behaviorally explicit questions in order to reduce variation in the interpretation and understanding of what violence comprises of. This tool simultaneously assesses women’s exposure to current and lifetime acts of IPV. We modified questions in the CTS2^68^ to assess maternal exposure to IPV subdivided by type (physical, sexual, and emotional abuse) at four time points: during pregnancy, within the first year of the child’s life, within the past 12 months (child’s second year of life), or at any point in their lifetime. Each woman was surveyed at Year 2 and asked to retrospectively recall their exposure for each time interval. Five behaviorally specific questions (regarding slapping, pushing, hitting with a fist, kicking, dragging, choking, burning, or threatening with a weapon) were asked for assessing physical IPV. A typical example included, ‘has your current or most recent husband slapped you or thrown something at you that could hurt you?’. Five questions (regarding forced sexual intercourse or other sexual acts) were used to measure sexual IPV. For example, ‘did your current or most recent husband ever physically force you to have sexual intercourse when you did not want to?’. Another four questions were asked for measuring emotional IPV (regarding insulting, humiliating, intimidating, threatening to hurt, etc.). A typical item was ‘has your current/most recent husband insulted you or made you feel bad about yourself?’. Each of the questions had ‘yes’ and ‘no’ response options for each time point. For each violence measure, a person was considered exposed if she responded ‘yes’ to any of the items related to that specific type of IPV and was coded ‘1 = Yes’, and ‘0 = No’ otherwise.

### Maternal depressive symptoms

We measured maternal depressive symptoms using the 20 question Center for Epidemiologic Studies Depression Scale Revised (CESD-20)^70^, a self-reported measure of depressive symptoms over the past 7 days. We used continuous CESD-20 scores at both Years 1 and 2, with possible scores ranging from 0 to 60 with higher scores representing more depressive symptoms. The United States-validated CESD-20 cutoff score of 16 has not been clinically validated in Bangladesh^70^. Therefore, we used continuous CESD-20 scores during the analysis. In addition to assessing CESD-20 scores as a continuous exposure, we measured CESD-20 scores as a binary exposure variable to provide a more clinically relevant assessment of depressive symptoms. Given the lack of a validated cutoff, the highest 25% of CESD-20 scores were classified as the “high depressive symptom” group and were compared to the remaining 75% who were classified as the “low depressive symptom group”.

### Parental perceived stress

At Year 2, we assessed parental stress using the Perceived Stress Scale (PSS)^71^, a 10-question survey that assesses an individual’s stress level over the past month. The questionnaire measures an individual’s subjective experience of stress. The possible range of scores is 0–40, with higher scores representing higher perceived stress.

### Outcomes

We collected blood samples from children at Year 1 (median age 14 months) and Year 2 (median age 28 months) to assess whole blood TL for all samples using quantitative polymerase chain reaction (qPCR) after sample collection in Year 2. The full protocol used for TL measurements was previously described^49^. We expressed relative TL as a ratio of telomere to single-copy gene abundance (T/S ratio). We transformed the TLs into Z-scores, which enables direct comparisons with other qPCR-based telomere studies^72^. We calculated Z-scores by first subtracting the average relative TL from the raw TL values, and then dividing by the standard deviation of relative TLs. TL Z-scores were standardized across the whole sample and calculated separately at Year 1 and Year 2. The final outcomes used in analysis were child TL Z-scores at Year 1, Year 2, and change in TL Z-scores between Year 1 and Year 2 (TL Z-score at Year 2 – TL Z-score at Year 1).

### Statistical analysis

We performed analyses for each exposure-outcome pair and used natural cubic splines to summarize the patterns and approximated Bayesian 95% confidence intervals around the fitted curves. We summarized the child TLs from the analyses, both unadjusted and adjusted for potential confounders, across the distributions of the exposures using natural smoothing cubic splines in generalized additive models. We used the code s(X, bs = “cr”) with the gam function in the mgcv package, which by default uses 10 knots. The 10 knots means 9 is the maximum possible degrees of freedom on the spline, but the actual degrees of freedom used is determined by the smoothing parameter (estimated by GCV/REML), so the effective degrees of freedom (edf) will be somewhere between 1 and k-1. When specifying bs = “cr”, the result is a cubic regression spline that is constrained to be linear beyond the boundary knots, which is equivalent to the natural boundary conditions. With the gam function, we used the identity link with the continuous telomere outcomes, so the model is equivalent to a multivariable linear regression but with spline terms instead of linear coefficients for all continuous predictors. We reported the adjusted difference and 95% confidence interval between the 25th versus 75th percentile of the distribution for each continuous exposure, or without exposure versus with exposure for each binary exposure, as detailed below. For each hypothesis, we also reported unadjusted p-values and p-values FDR-corrected using the Benjamini-Hochberg procedure. The unadjusted and corrected p-values are listed in Supplementary Tables 1–3. Given the exploratory nature of our analyses, in addition to the typical FDR corrections to assess the probability of Type I error, we also evaluated whether multiple measures of a related exposure-outcome domain (e.g., multiple time points of maternal exposure to IPV and child TL) were consistent in magnitude and direction with each other. Thus, if relationships within one domain of exposure-outcome clustered closely above and below the null hypothesis, we concluded that a standalone statistically significant result in that cluster may have been spurious. In contrast, if relationships within one domain of exposure-outcome clustered closely to one another but away from the null hypothesis in a consistent direction, we interpreted this consistency in observations as a result of an underlying relationship rather than chance.

In all analyses, we adjusted for child age, sex, and a pre-screened list of covariates found to be significantly related to the outcome. The maternal and household covariates were measured at enrollment, while child covariates were measured at ages 3, 14, or 28 months. The full list of potential covariates, included in the pre-registered analysis plan on Open Science Framework (https://osf.io/q4fmu/), were selected based on possible confounders in the existing literature (Supplementary Fig. 2). All analyses were adjusted for the following covariates: child sex, child birth order, mother’s age, mother’s height, mother’s education, household food security, number of children < 18 years old in the household, number of people living in the compound, month of exposure and outcome measurement, treatment arm, distance (in minutes) to the primary water source, household materials (wall, floor, roof), asset-based household wealth (electricity, wardrobe, table, chair or bench, khat, chouki, working radio, working black/white or color television, refrigerator, bicycle, motorcycle, sewing machine, mobile phone, land phone, number of cows, number of goats, number of chickens). We also tested for characteristics measured during follow-up, including monsoon season indicators, child age, and treatment arm status. We performed the likelihood ratio test on each outcome-covariate pair and included all covariates with a p-value < 0.2.

All statistical analysis was performed using R statistical software version 4.0.4.

### Post-hoc analyses

We calculated Pearson correlation coefficients for pairwise complete observations for maternal IPV, maternal depressive symptoms, and parental perceived stress as well as their correlations with child TL.

As physical and emotional IPV are more likely to be witnessed by children and impact their TL differentially compared to sexual IPV, we conducted secondary analyses with exposures of maternal lifetime exposure to each type of IPV (physical, emotional, or sexual) versus child TL outcomes. As an additional secondary analysis to investigate potentially distinct associations between varying degrees of IPV and child TL, we estimated associations between frequency of types of IPV and telomere outcomes using covariate-adjusted multivariate regression models, where separate models were fit contrasting each frequency (once, several times, most/all the time) against a reference level of never.

## Electronic supplementary material

Below is the link to the electronic supplementary material.

Supplementary Material 1

## Data Availability

All data and R scripts are publicly available on Open Science Framework (https://osf.io/f2cm5/).
