# Supplementary material for: Maternal experience of intimate partner violence, maternal depression, and parental stress are not associated with child telomere length in Bangladesh
Source: Sci Rep. 2025 Mar 12;15:8499. doi: 10.1038/s41598-025-90505-2 (PMC11903653; doi:10.1038/s41598-025-90505-2)
Supplement: Supplementary file 1 — Supplementary Material 1 [file 41598_2025_90505_MOESM1_ESM.docx]

**Supplementary File**

**Maternal intimate partner violence, depression, and parental perceived stress are not associated with young child telomere length in Bangladesh**

Diego Figueroa, Md. Mahfuz Al Mamun, Da Kyung Jung, Gaoge Li, Sophia T. Tan, Farheen Jamshed, Zachary Butzin-Dozier, Andrew N. Mertens, Jue Lin, Helen O. Pitchik, Kausar Parvin, Alexis Silvera, Lia C. H. Fernald, Benjamin F. Arnold, Shahjahan Ali, Abul K. Shoab, Syeda Luthfa Famida, Salma Akther, Md. Ziaur Rahman, Md. Saheen Hossen, Palash Mutsuddi, Mahbubur Rahman, Leanne Unicomb, Patricia Kariger, Christine P. Stewart, Alan E. Hubbard, Jade Benjamin-Chung, Firdaus S. Dhabhar, Stephen P. Luby, John M. Colford Jr., Ruchira T. Naved, Audrie Lin

**
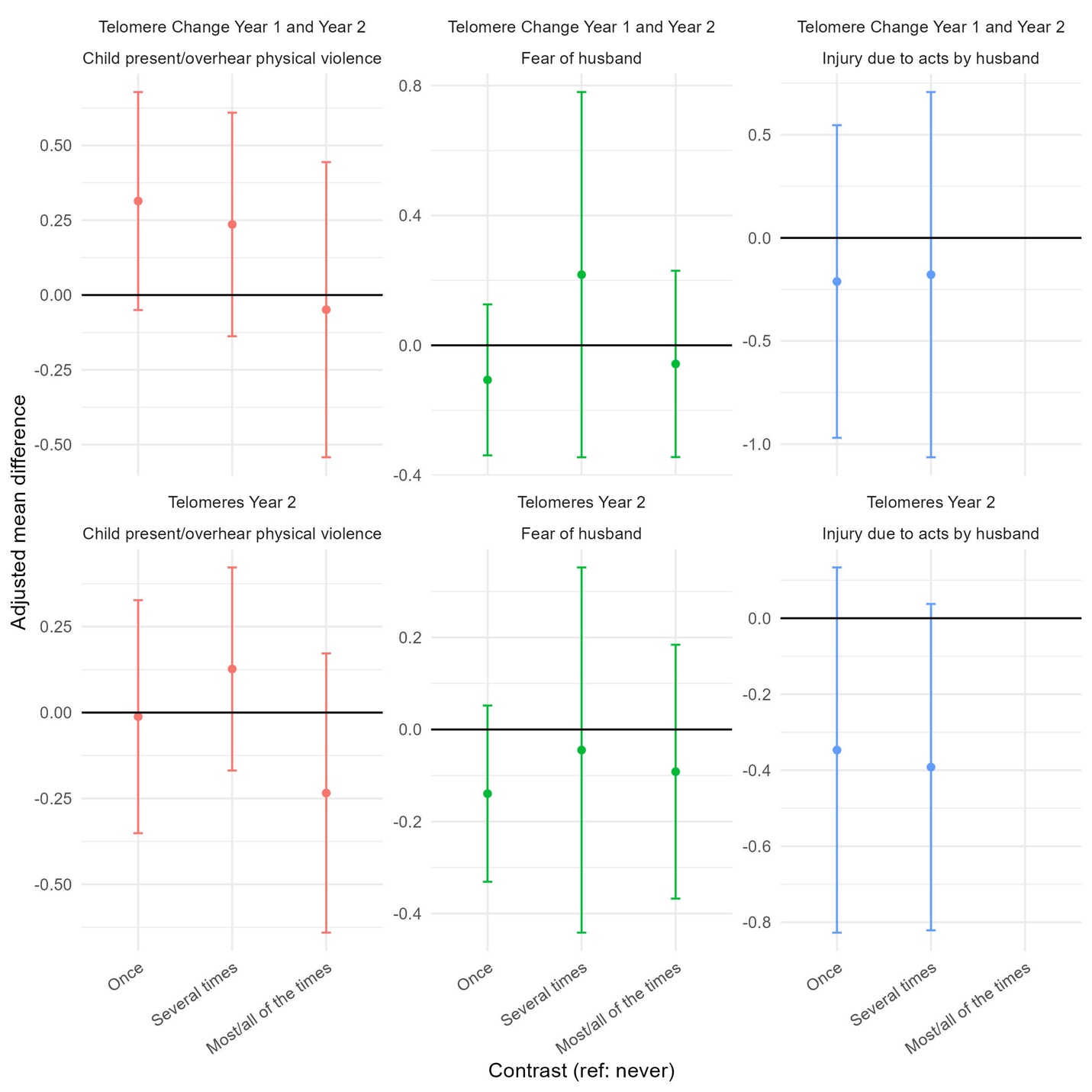
Supplementary Figure 1: A visual summary of the associations between varying frequencies of maternal exposure to intimate partner violence and child telomere length**


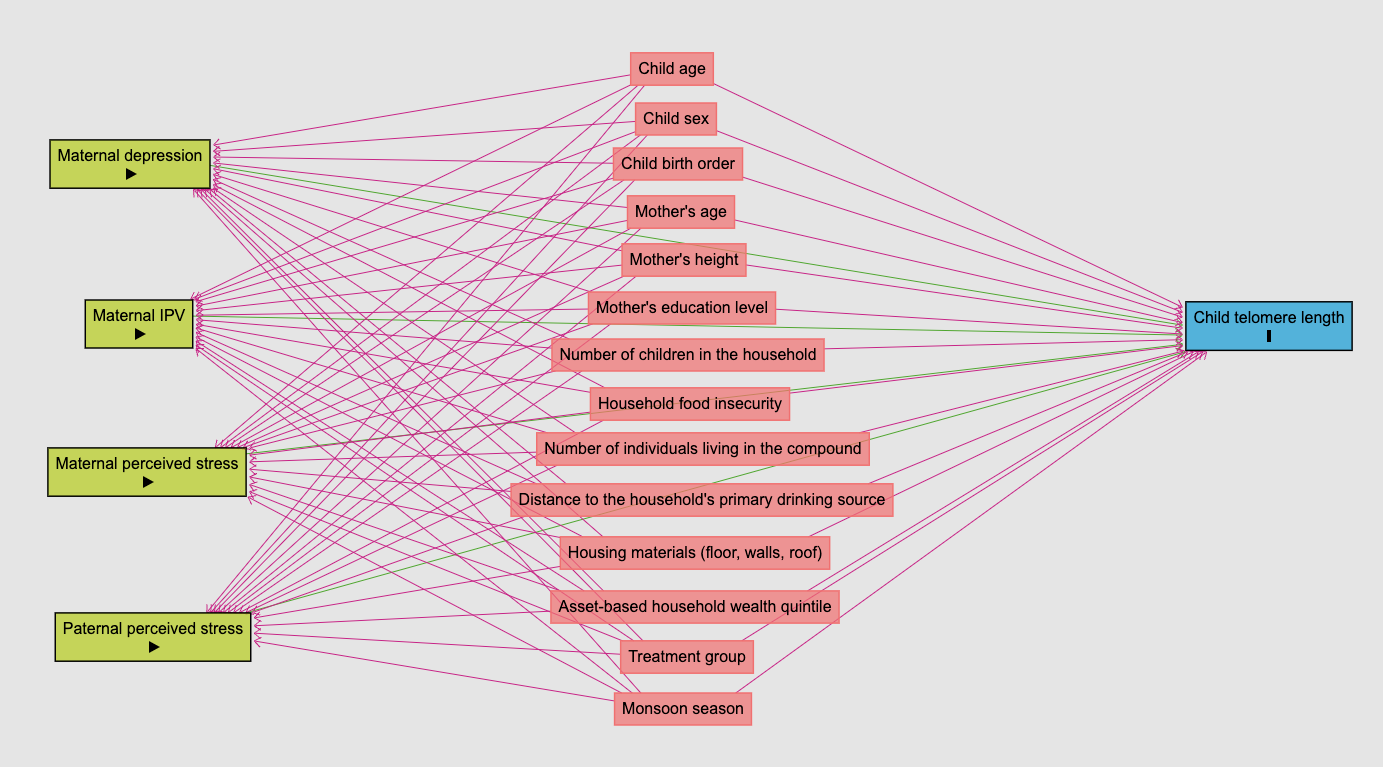


**Supplementary Figure 2: A Directed Acyclic Graph (DAG) visualizing potential causal paths between the parental exposures, child telomere length, and covariates**

**Supplementary Table 1: Unadjusted and Adjusted Associations Between Maternal Exposure to IPV and Child Telomere Length**

| IPV | Outcome | N | Outcome, With Exposure v. Without Exposure | | | | | | | | | | |
| --- | --- | --- | --- | --- | --- | --- | --- | --- | --- | --- | --- | --- | --- |
|  |  |  | Unadjusted | | | | | Adjusted^*^ | | | | | |
|  |  |  |  | | | | |  | | | | | |
|  |  |  | Predicted Outcome if Not Exposed | Predicted Outcome if Exposed | Predicted Outcome Difference (95% CI) | P-value | FDR Corrected P-value | Predicted Outcome if Not Exposed | Predicted Outcome if Exposed | Predicted Outcome Difference (95% CI) | P-value | FDR Corrected P-value | |
| Lifetime Exposure to IPV Year 2 | Telomere length Z-score Year 2 | 686 | -0.03 | -0.14 | -0.11 (-0.26, 0.04) | 0.14 | 0.26 | 0.3 | 0.17 | -0.13 (-0.28, 0.02) | 0.08 | 0.33 | |
|  |  |  |  |  |  |  |  |  |  |  |  |  | |
| Exposure to IPV during Pregnancy | Telomere length Z-score Year 1 | 300 | 0 | -0.07 | -0.07 (-0.36, 0.22) | 0.63 | 0.79 | 0 | -0.13 | -0.13 (-0.43, 0.17) | 0.41 | 0.6 | |
|  | Telomere length Z-score Year 2 | 367 | -0.03 | -0.29 | -0.25 (-0.49, -0.01) | 0.04 | 0.12 | 0.03 | -0.16 | -0.19 (-0.43, 0.05) | 0.13 | 0.33 | |
|  | Change in Telomere length Z-Score | 288 | 0.08 | -0.26 | -0.33 (-0.63, -0.03) | 0.03 | 0.12 | 0.26 | -0.06 | -0.32 (-0.64, -0.01) | 0.05 | 0.33 | |
|  |  |  |  |  |  |  |  |  |  |  |  |  | |
| Exposure to IPV between birth and Year 1 | Telomere length Z-score Year 1 | 312 | -0.04 | -0.01 | 0.03 (-0.2, 0.27) | 0.79 | 0.79 | -0.07 | -0.08 | 0 (-0.25, 0.25) | 0.99 | 0.99 | |
|  | Telomere length Z-score Year 2 | 383 | -0.09 | -0.28 | -0.19 (-0.39, 0.01) | 0.07 | 0.15 | -0.11 | -0.25 | -0.14 (-0.35, 0.07) | 0.2 | 0.36 | |
|  | Change in Telomere length Z-Score | 297 | 0.03 | -0.22 | -0.26 (-0.49, -0.02) | 0.03 | 0.12 | 0.26 | 0.06 | -0.19 (-0.45, 0.07) | 0.15 | 0.33 | |
|  |  |  |  |  |  |  |  |  |  |  |  |  | |
| Exposure to IPV between Year 1 and Year 2 | Telomere length Z-score Year 2 | 383 | -0.04 | -0.17 | -0.13 (-0.34, 0.08) | 0.22 | 0.33 | -0.06 | -0.14 | -0.08 (-0.3, 0.13) | 0.47 | 0.6 | |
|  | Change in Telomere length Z-Score | 297 | 0 | -0.04 | -0.04 (-0.29, 0.21) | 0.77 | 0.79 | 0.17 | 0.18 | 0.01 (-0.24, 0.27) | 0.94 | 0.99 | |
| N, 25th Percentile, and 75th Percentile are from the unadjusted generalized additive model analyses  * Adjusted for pre-specified and pre-screened covariates: child sex, child birth order, mother’s age, mother’s height, mother’s education, household food security, number of children < 18 years old in the household, number of people living in the compound, month of exposure and outcome measurement, treatment arm, distance (in minutes) to the primary water source, household materials (wall, floor, roof), asset-based household wealth (electricity, wardrobe, table, chair or bench, khat, chouki, working radio, working black/white or color television, refrigerator, bicycle, motorcycle, sewing machine, mobile phone, land phone, number of cows, number of goats, number of chickens). | | | | | | | | | | | | |  |

## **Supplementary Table 2: Unadjusted and Adjusted Associations Between Maternal Depression and Child Telomere Length**

| Maternal Depression | Outcome | N | 25th Percentile | 75th Percentile | Outcome, 75th Percentile v. 25th Percentile or With Exposure v. Without Exposure | | | | | | | | | |
| --- | --- | --- | --- | --- | --- | --- | --- | --- | --- | --- | --- | --- | --- | --- |
|  |  |  |  |  | Unadjusted | | | | | Adjusted^*^ | | | | |
|  |  |  |  |  | Predicted Outcome at 25th Percentile or if Not Exposed | Predicted Outcome at 75th Percentile or if Exposed | Predicted Outcome Difference (95% CI) | P-value | FDR Corrected P-value | Predicted Outcome at 25th Percentile or if Not Exposed | Predicted Outcome at 75th Percentile or if Exposed | Predicted Outcome Difference (95% CI) | P-value | FDR Corrected P-value |
| Continuous Maternal Depressive Symptoms Year 1 | Telomere length Z-score Year 1 | 658 | 6 | 16 | -0.04 | 0.03 | 0.07 (-0.03, 0.17) | 0.17 | 0.65 | 0.05 | 0.12 | 0.07 (-0.03, 0.16) | 0.18 | 0.57 |
|  | Telomere length Z-score Year 2 | 699 | 6 | 16 | -0.13 | -0.09 | 0.04 (-0.05, 0.13) | 0.41 | 0.66 | 0.2 | 0.25 | 0.05 (-0.04, 0.13) | 0.29 | 0.57 |
|  | Change in Telomere length Z-Score | 555 | 6 | 16 | -0.11 | -0.13 | -0.02 (-0.12, 0.08) | 0.69 | 0.69 | 0.26 | 0.25 | -0.01 (-0.11, 0.1) | 0.87 | 0.87 |
|  |  |  |  |  |  |  |  |  |  |  |  |  |  |  |
| Binary Maternal Depressive Symptoms Year 1 | Telomere length Z-score Year 1 | 658 | -- | -- | -0.04 | 0.11 | 0.15 (-0.02, 0.32) | 0.09 | 0.65 | 0.06 | 0.17 | 0.11 (-0.06, 0.28) | 0.22 | 0.57 |
|  | Telomere length Z-score Year 2 | 699 | -- | -- | -0.13 | -0.04 | 0.09 (-0.08, 0.25) | 0.32 | 0.65 | 0.21 | 0.31 | 0.1 (-0.06, 0.27) | 0.22 | 0.57 |
|  | Change in Telomere length Z-Score | 555 | -- | -- | -0.11 | -0.15 | -0.04 (-0.23, 0.15) | 0.67 | 0.69 | 0.26 | 0.24 | -0.02 (-0.21, 0.17) | 0.82 | 0.87 |
|  |  |  |  |  |  |  |  |  |  |  |  |  |  |  |
| Continuous Maternal Depressive Symptoms Year 2 | Telomere length Z-score Year 2 | 687 | 5 | 17 | -0.11 | -0.09 | 0.02 (-0.06, 0.11) | 0.61 | 0.69 | 0.19 | 0.22 | 0.03 (-0.06, 0.11) | 0.57 | 0.76 |
|  |  |  |  |  |  |  |  |  |  |  |  |  |  |  |
| Binary Maternal Depressive Symptoms Year 2 | Telomere length Z-score Year 2 | 687 | -- | -- | -0.12 | -0.03 | 0.09 (-0.08, 0.26) | 0.28 | 0.65 | 0.19 | 0.27 | 0.08 (-0.09, 0.24) | 0.36 | 0.58 |
| N, 25th Percentile, and 75th Percentile are from the unadjusted generalized additive model analyses  * Adjusted for pre-specified and pre-screened covariates: child sex, child birth order, mother’s age, mother’s height, mother’s education, household food security, number of children < 18 years old in the household, number of people living in the compound, month of exposure and outcome measurement, treatment arm, distance (in minutes) to the primary water source, household materials (wall, floor, roof), asset-based household wealth (electricity, wardrobe, table, chair or bench, khat, chouki, working radio, working black/white or color television, refrigerator, bicycle, motorcycle, sewing machine, mobile phone, land phone, number of cows, number of goats, number of chickens). | | | | | | | | | | | | | | |

##

**Supplementary Table 3: Unadjusted and Adjusted Associations Between Parental Stress and Child Telomere Length**

| Parental Stress | Outcome | N | 25th Percentile | 75th Percentile | Outcome, 75th Percentile v. 25th Percentile | | | | | | | | | |
| --- | --- | --- | --- | --- | --- | --- | --- | --- | --- | --- | --- | --- | --- | --- |
|  |  |  |  |  | Unadjusted | | | | | Adjusted^*^ | | | | |
|  |  |  |  |  | Predicted Outcome at 25th Percentile | Predicted Outcome at 75th Percentile | Predicted Outcome Difference (95% CI) | P-value | FDR Corrected P-value | Predicted Outcome at 25th Percentile | Predicted Outcome at 75th Percentile | Predicted Outcome Difference (95% CI) | P-value | FDR Corrected P-value |
| Maternal Perceived Stress | Telomere Length Z-score Year 2 | 688 | 10 | 18 | -0.08 | -0.1 | -0.02 (-0.13, 0.09) | 0.76 | 0.76 | 0.21 | 0.18 | -0.04 (-0.14, 0.07) | 0.51 | 0.51 |
|  |  |  |  |  |  |  |  |  |  |  |  |  |  |  |
| Paternal Perceived Stress | Telomere Length Z-score Year 2 | 502 | 14 | 21 | -0.22 | -0.07 | 0.15 (-0.04, 0.34) | 0.13 | 0.26 | 0.2 | 0.33 | 0.13 (-0.05, 0.32) | 0.16 | 0.32 |
| N, 25th Percentile, and 75th Percentile are from the unadjusted generalized additive model analyses  * Adjusted for pre-specified and pre-screened covariates: child sex, child birth order, mother’s age, mother’s height, mother’s education, household food security, number of children < 18 years old in the household, number of people living in the compound, month of exposure and outcome measurement, treatment arm, distance (in minutes) to the primary water source, household materials (wall, floor, roof), asset-based household wealth (electricity, wardrobe, table, chair or bench, khat, chouki, working radio, working black/white or color television, refrigerator, bicycle, motorcycle, sewing machine, mobile phone, land phone, number of cows, number of goats, number of chickens). | | | | | | | | | | | | | | |

**Supplementary Table 4: Adjusted Associations Between Maternal Exposure to Each Type of IPV and Child Telomere Length**

| Type of IPV | Outcome | N | 25th Percentile | 75th Percentile | Outcome, 75th Percentile v. 25th Percentile | | | |
| --- | --- | --- | --- | --- | --- | --- | --- | --- |
|  |  |  |  |  | Adjusted | | | |
|  |  |  |  |  | Predicted Outcome at 25th Percentile | Predicted Outcome at 75th Percentile | Predicted Outcome Difference (95% CI) | P-value |
| Lifetime Exposure to Emotional Violence Year 2 | Telomere length Z-score Year 1 | 541 | -- | -- | 0.04 | 0.05 | 0 (-0.17, 0.18) | 0.96 |
|  | Telomere length Z-score Year 2 | 678 | -- | -- | 0.23 | 0.19 | -0.04 (-0.19, 0.11) | 0.6 |
|  | Change in Telomere length Z-Score | 524 | 0 | 1 | 0.26 | 0.26 | -0.01 (-0.18, 0.17) | 0.95 |
|  |  |  |  |  |  |  |  |  |
| Lifetime Exposure to Physical Violence Year 2 | Telomere length Z-score Year 1 | 541 | 0 | 1 | 0.09 | 0 | -0.09 (-0.26, 0.07) | 0.27 |
|  | Telomere length Z-score Year 2 | 678 | 0 | 1 | 0.28 | 0.16 | -0.12 (-0.27, 0.03) | 0.12 |
|  | Change in Telomere length Z-Score | 524 | 0 | 1 | 0.29 | 0.22 | -0.07 (-0.24, 0.1) | 0.41 |
|  |  |  |  |  |  |  |  |  |
| Lifetime Exposure to Sexual Violence Year 2 | Telomere length Z-score Year 1 | 541 | 0 | 1 | 0.04 | 0.05 | 0 (-0.2, 0.2) | 0.98 |
|  | Telomere length Z-score Year 2 | 678 | 0 | 1 | 0.27 | 0.12 | -0.15 (-0.33, 0.02) | 0.09 |
|  | Change in Telomere length Z-Score | 524 | 0 | 1 | 0.27 | 0.22 | -0.05 (-0.25, 0.16) | 0.65 |
| N, 25th Percentile, and 75th Percentile are from the adjusted analyses  Adjusted for pre-specified and pre-screened covariates: child sex, child birth order, mother’s age, mother’s height, mother’s education, household food security, number of children < 18 years old in the household, number of people living in the compound, month of exposure and outcome measurement, treatment arm, distance (in minutes) to the primary water source, household materials (wall, floor, roof), asset-based household wealth (electricity, wardrobe, table, chair or bench, khat, chouki, working radio, working black/white or color television, refrigerator, bicycle, motorcycle, sewing machine, mobile phone, land phone, number of cows, number of goats, number of chickens). | | | | | | | | |

**Supplementary Table 5: Correlations Between Maternal IPV, Maternal Depressive Symptoms, and Parental Perceived Stress, and Their Correlations with Child Telomere Length**

| **X** | **Y** | **Pearson Correlation Coefficient** |
| --- | --- | --- |
| Lifetime maternal IPV | Maternal depression (CESD) | 0.200 |
| Lifetime maternal IPV | Maternal stress (PSS) | 0.100 |
| Lifetime maternal IPV | Paternal stress (PSS) | 0.109 |
| Maternal depression (CESD) | Maternal stress (PSS) | 0.382 |
| Maternal depression (CESD) | Paternal stress (PSS) | 0.019 |
| Lifetime maternal IPV | Change in Telomere Length | -0.048 |
| Maternal depressive symptoms (CESD) | Change in Telomere Length | -0.123 |
| Lifetime maternal IPV | Telomere Length - Year 1 | -0.042 |
| Maternal depressive symptoms (CESD) | Telomere Length - Year 1 | 0.082 |
| Lifetime maternal IPV | Telomere Length - Year 2 | -0.054 |
| Maternal depressive symptoms (CESD) | Telomere Length - Year 2 | -0.025 |
| Maternal stress (PSS) | Telomere Length - Year 2 | -0.009 |
| Paternal stress (PSS) | Telomere Length - Year 2 | 0.040 |
